# *Staphylococcus aureus* mediates pyroptosis in bovine mammary epithelial cell via activation of NLRP3 inflammasome

**DOI:** 10.1186/s13567-022-01027-y

**Published:** 2022-02-05

**Authors:** Xiaozhou Wang, Mingchao Liu, Na Geng, Yongzhen Du, Zhaoming Li, Xin Gao, Bo Han, Jianzhu Liu, Yongxia Liu

**Affiliations:** 1 College of Veterinary Medicine, Shandong Agricultural University, Tai’an, 271018 Shandong China; 2 College of Veterinary Medicine, Hebei Agricultural University, Baoding, 071001 Hebei China; 3 Research Center for Animal Disease Control Engineering, Shandong Agricultural University, Tai’an, 271018 Shandong China; 4 College of Veterinary Medicine, China Agricultural University, Beijing, 100193 China

**Keywords:** *Staphylococcus aureus*, bovine mastitis, inflammasome, caspase-1, pyroptosis

## Abstract

Cell death and inflammation are intimately linked during mastitis due to *Staphylococcus aureus* (*S. aureus*). Pyroptosis, a programmed necrosis triggered by gasdermin protein family, often occurs after inflammatory caspase activation. Many pathogens invade host cells and activate cell-intrinsic death mechanisms, including pyroptosis, apoptosis, and necroptosis. We reported that bovine mammary epithelial cells (MAC-T) respond to *S. aureus* by NOD-like receptor protein 3 (NLRP3) inflammasome activation through K^+^ efflux, leading to the recruitment of apoptosis-associated speck-like protein (ASC) and the activation of caspase-1. The activated caspase-1 cleaves gasdermin D (GSDMD) and forms a N-terminal pore forming domain that drives swelling and membrane rupture. Membrane rupture results in the release of the pro-inflammatory cytokines IL-18 and IL-1β, which are activated by caspase-1. Can modulate GSDMD activation by NLRP3-dependent caspase-1 activation and then cause pyroptosis of bovine mammary epithelial cells.

**Supplementary Information:**

The online version contains supplementary material available at 10.1186/s13567-022-01027-y.

## Introduction

*Staphylococcus aureus* infection of the udder in dairy herds is a major cause of bovine mastitis. *Staphylococcus aureus* is always an important problem to the dairy industry worldwide because of its contagiousness, pathogenicity, and poor prognosis; it also poses a threat to food safety and public health [1, 2]. *Staphylococcus aureus* is widespread in the natural environment of dairy farms; its adhesion and invasion of epithelial cells has made the treatment of mastitis difficult [3]. Sensing of *S. aureus* in bovine mammary glands involves epithelial cells that trigger a cascade of immunity-related processes to kill or inactivate *S. aureus* [4].

Pyroptosis is an inflammatory form of cell death caused by inflammasomes that can be triggered by a variety of stimuli, including bacterial infection and danger signals [5]. GSDMD of the gasdermin protein family is cleaved by inflammatory caspases and exhibits pore forming activity to drive pyroptosis [6, 7]. Inflammasomes play an important role in innate immunity, among which the NLRP3 inflammasome is the best characterized one; NLRP3 can be activated by bacteria through K^+^ efflux [8, 9]. NLRP3 inflammasomes is an intracellular supramolecular complex composed of the sensor molecules NLRP3, ASC, and caspase-1 [10]. Upon the activation of inflammasome sensor molecules, ASC oligomerizes, thereby forming ASC specks [11]. Full-length caspase-1 is subsequently recruited to the inflammasome and activated by self-cleavage upon interaction with ASC [12]. Upon activation by inflammasomes, caspase-1 can cleave pro-inflammatory cytokines IL-1β and IL-18 into mature forms. Activated caspase-1 can also cleave GSDMD to form GSDMD-N, which binds to the plasma membrane and generates membrane pores, leading to the release of mature IL-1 β and IL-18, cell swelling, and eventually, lysis [13, 14].

Pyroptosis may be one of the important mechanisms underlying the pathogenesis of bovine mastitis. In this work, we studied whether *S. aureus* can induce pyroptosis in MAC-T cells and further explored its detailed mechanism.

## Materials and methods

### Bacterial strains and culture conditions

*Staphylococcus aureus* strain ATCC25178 was isolated from bovine mastitis. First, the bacteria were inoculated at the Luria–Bertani (LB) Agar at 37 °C. After 24 h, a separate colony was randomly selected, placed in LB broth, and placed on a shaker at 200 rpm and 37 °C. After 12 h of culture, bacterial growth was monitored by measuring the OD_600nm_.

### Cell culture and treatment

MAC-T cells were cultured at 37 °C with 5% CO_2_ in Dulbecco’s modified Eagle’s medium (Gibco, USA) supplemented with 10% fetal bovine serum (FBS, Biological Industries, Israel). When cultured to monolayers, cells were infected at a multiplicity of infection (MOI) of 15 in the culture medium. After 2 h of infection, infected cells were washed thrice with PBS, incubated in medium containing lysostaphin (10 μg/mL) and gentamicin (100 μg/mL) to kill extracellular bacteria for 15 min, and incubated in medium containing gentamicin (50 μg/mL) to limit the extracellular replication of *S. aureus*.

### Preparation of antibody and inhibitors

The GSDMD-N antibody preparation was completed by ABclonal Co., Ltd. (China). Rabbits were immunized with the peptide to obtain serum. The peptide sequence used for antibody preparation is shown in Additional file 1. Then, the specific antibodies were obtained by affinity purification of the antigen. Caspase-1 inhibitor VX765 and NLRP3 inflammasome inhibitor MCC950 were obtained from Glpbio (USA) and used at final concentrations of 100 and 15 µM.

### Measurement of cytokines and activated caspase-1

Cytokine levels in cell culture supernatants were determined by ELISAs for bovine IL-1β (Raybiotech, USA) and IL 18 (ABclonal, China). Caspase-1 activity in MAC-T cells was measured using a commercial Caspase-1 Activity Assay Kit (Abbkine, China). All steps were performed according to the manufacturer’s guidelines. This assay was based on the hydrolysis of the peptide substrate acetyl-Tyr-Val-Ala-Asp p-nitroanilide (Ac-YVAD-pNA) by caspase-1, which resulted in the release of the yellow formazan product p-nitroaniline (pNA). After treatment, cells were lysed and harvested. Then, they were incubated at 37 °C for 2 h with PNA Ac-YVAD-pNA to produce the yellow formazan product pNA, which can be quantified at 405 nm by spectrophotometer. Caspase-1 activity was obtained by determining the amount of pNA in the sample according to the standard curve of pNA.

### Cytotoxicity and cell death assays

For cytotoxicity assays, cells grown on coverslips were treated with *S. aureus* for 4 h. Cytotoxicity was assessed by double staining cells with annexin V-FITC/Propidium iodide (PI, Elabscience, China). Annexin V binding requires media supplemented up to 2 mM CaCl_2_ and controlled at pH 7.2–7.5. The LDH (lactate dehydrogenase) assay (Dojindo, Japan) was used to measure cell death by cell culture supernatants.

### Immunoblotting

At the desired time points post infection or stimulation, cells were lysed in RIPA lysis buffer (Ncmbio, China) with protease inhibitors on ice for 10 min. The cells were then centrifuged at 12 000 × *g* for 10 min, and the supernatant was obtained and then denatured in 1X Laemmli Buffer with 5% β-mercaptoethanol. Proteins were separated by 10%–15% SDS–PAGE gel and transferred to PVDF membranes (Merck Millipore, USA). Then, they were blocked with PBST and 5% skim milk for 1 h at RT. After washing with PBST, the membranes were incubated overnight at 4 °C with mouse anti-Caspase-1 (22915-1-AP, Proteintech, USA), rabbit anti-GSDMD (DF12275, Affbiotech, China), rabbit anti-IL-1β (GTX55675, GeneTex, USA), or mouse anti-β-actin (66009-1-Ig, Proteintech, USA). After washing with PBST, membranes were incubated with HRP-conjugated secondary antibodies. Proteins were visualized with ECL detection kit (Clinx, China).

### Fluorescence and confocal microscopy

To examine the ASC speck formation or GSDMD-N aggregation on the cell membrane, MAC-T cells grown on coverslips were treated with or without *S. aureus* for 4 h, fixed in 4% paraformaldehyde, permeabilized with Triton X-100, and blocked with 10% BSA. Subsequently cells were incubated with antibodies against ASC (10500-1-AP, Proteintech, USA), GSDMD (DF12275, Affbiotech, China) or GSDMD-N and stained with Alexa Fluor^®^ 488 or Alexa Fluor^®^ 647 secondary antibody. Nuclei were stained with Hoechst 33342 (Beyotime, China).

### Statistical analysis

Each experiment was independently repeated at least thrice. Experimental data were analyzed using GraphPad Prism 8. The statistical significance of differences between groups were analyzed by one-way ANOVA with Dunnett’s multiple comparison test. *P* values of less than 0.05 were considered significant and designated by: *P < 0.05, ** *P* < 0.01.

## Results

### *Staphylococcus aureus* induces GSDMD cleavage to drive pyroptosis

**Figure 1:**
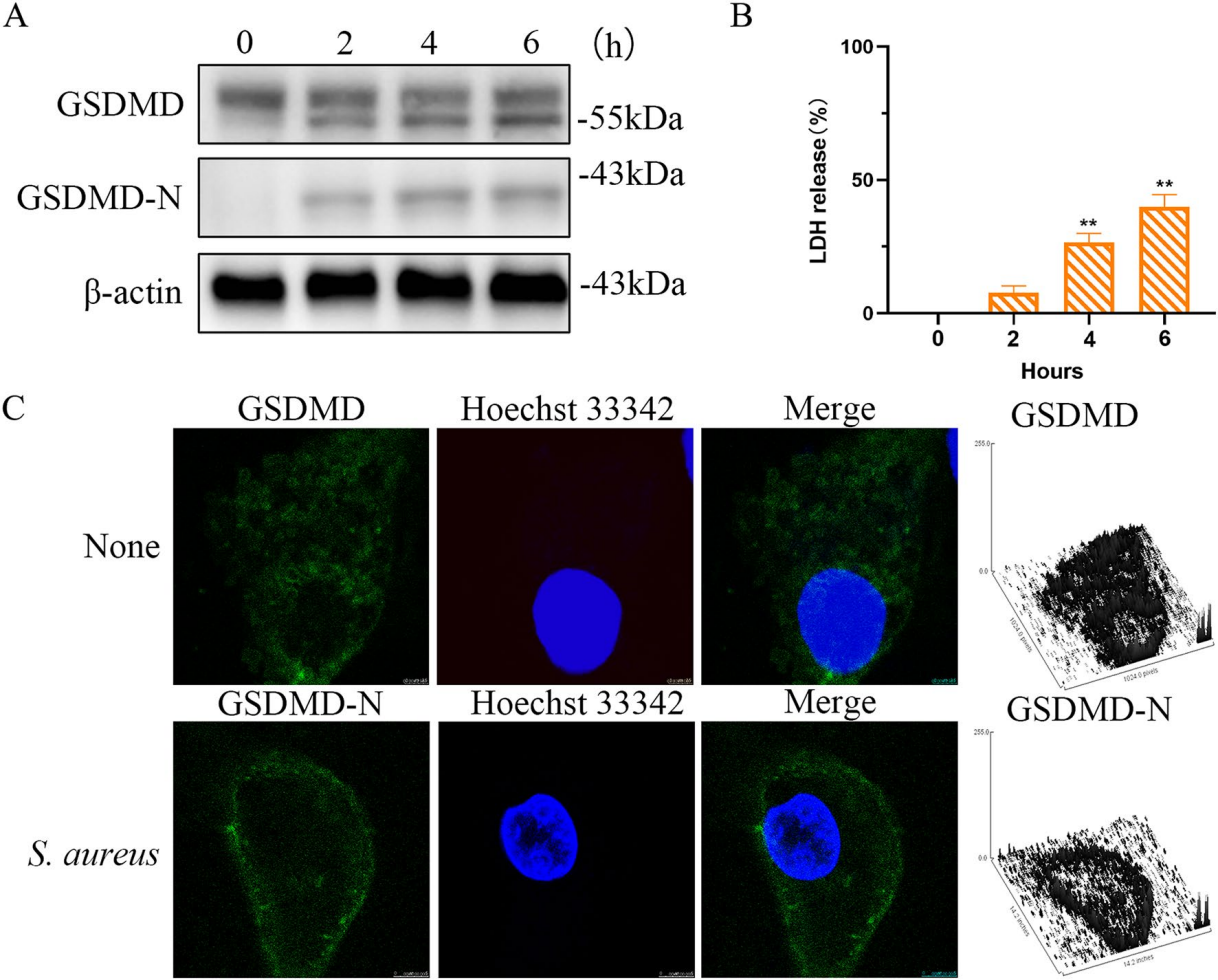
**GSDMD activation by *S. aureus*. A** MAC-T cells were treated with *S. aureus* for the indicated times**.** GSDMD and GSDMD-N were detected by immunoblotting. **B** Cell death was measured by LDH release. **C** Subcellular localization of GSDMD-N or GSDMD in MAC-T cells incubated with or without *S. aureus.*

GSDMD is a pyroptosis effector downstream of caspase activation; GSDMD-N is an executor of pyroptosis [15]. To determine whether GSDMD is involved in *S. aureus*-induced cell death, we stimulated MAC-T cells with *S. aureus* and examined the active cleavage products of GSDMD in cell lysates. GSDMD was cleaved upon stimulation by *S. aureus* (Figure 1A). Cell death was presented as the percentage of the LDH measured in the culture medium (Figure 1B). GSDMD uniformly distributed in cytoplasm in the absence of *S. aureus* stimulation. GSDMD-N was translocated and accumulated in the plasma membrane after 2 h of *S. aureus* stimulation as pyroptosis progressed (Figure 1C).

### *S. aureus*-driven pyroptosis by morphology

**Figure 2:**
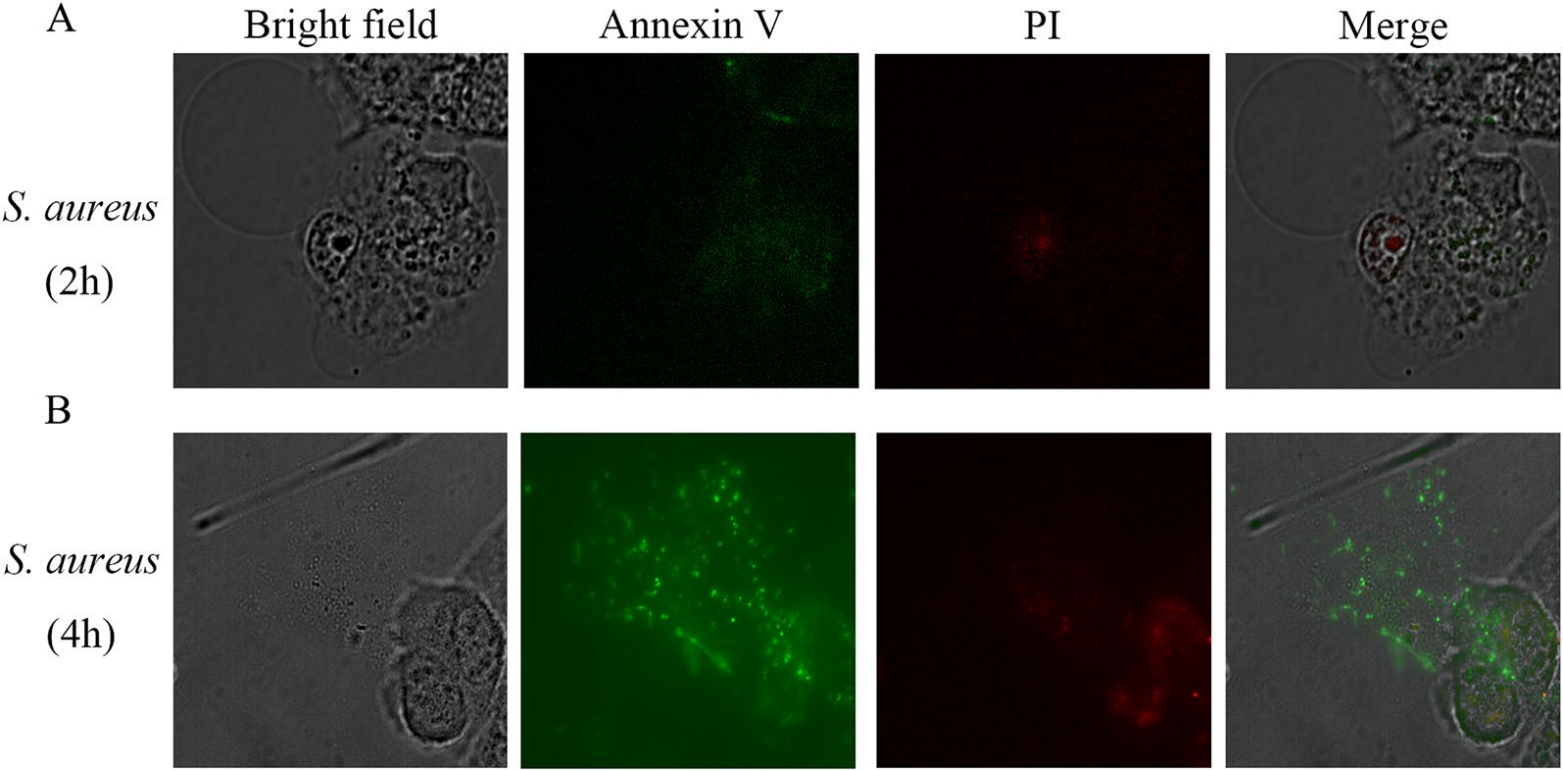
** Staphylococcus aureus driven cell death resembles pyroptosis by morphology.** **A** Fluorescence microscopy images of cells stained with annexin V and PI in MAC-T cells treated with *S. aureus* for 2 h and **B** 4 h.

Pyroptosis is a necrotic form of cell death, in which cell membranes rupture during pyroptosis [16]. Annexin V interacts with the phospholipid phosphatidylserine (PS), and when phosphatidylserine is translocated to the outer leaflet of the plasma membrane, annexin V binds to it and stains it. Considering that pyroptotic cells also have ruptured membranes, annexin V also stains the plasma membrane. PI is a DNA dye that does not permeate cell membranes, and DNA can be stained by PI after cell membrane rupture. As shown in Figure 2A, cells were markedly swollen, and characteristic large bubbles appeared in the plasma membrane; these are among the typical features of pyroptosis [17]. Membrane disruption and PI signaling were observed in MAC-T cells infected with S. aureus for 4 h (Figure 2B).

### *Staphylococcus aureus* activates caspase-1 and induces the release of IL-1β and IL-18

**Figure 3:**
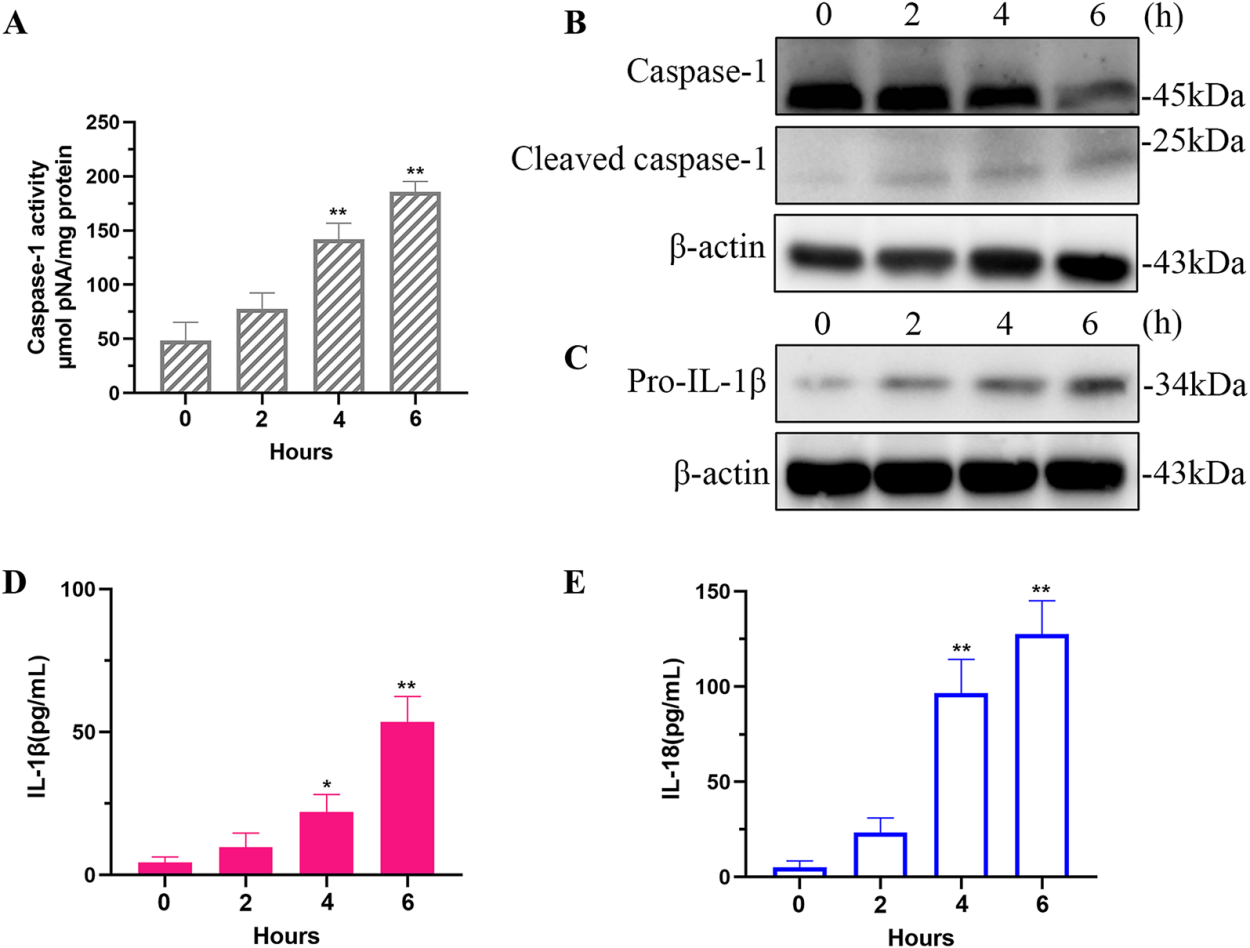
**Caspase-1 and cytokine activation by *S. aureus*** **. A** Detection of activated caspase-1 in MAC-T cells treated with *S. aureus* for the indicated times. **B** Cleaved caspase-1 and **C** pro-IL-1β were detected by immunoblotting. **D** Time course of IL-1β and IL-18 production by MAC-T cells treated with *S. aureus.*

During pyroptosis, inflammasomes can cleave pro-caspase-1 to generate cleaved caspase-1. Cleaved caspase-1 can directly cleave GSDMD to generate GSDMD-N and can also cleave pro-IL-1 β and pro-IL-18 to produce the mature cytokines IL-1 β and IL-18. The mature IL-1 β and IL-18 are subsequently released from the plasma membrane pores formed by GSDMD-N [18]. The release of IL-1β and IL-18 depends on caspase-1-mediated cleavage. Thus, we measured caspase-1 activity in MAC-T supernatants (Figure 3A). Results of immunoblotting experiments showed that after *S. aureus* infection of MAC-T cells, the expressions of cleaved caspase-1 and pro-IL-1β began to increase (Figures 3B and C). In time course experiments, secretions of IL-1β and IL-18 by MAC-T cells were detected as early as 4 h after treatment with *S. aureus* (Figures 3D and E).

### ASC speck formation mediated by NLRP3

**Figure 4:**
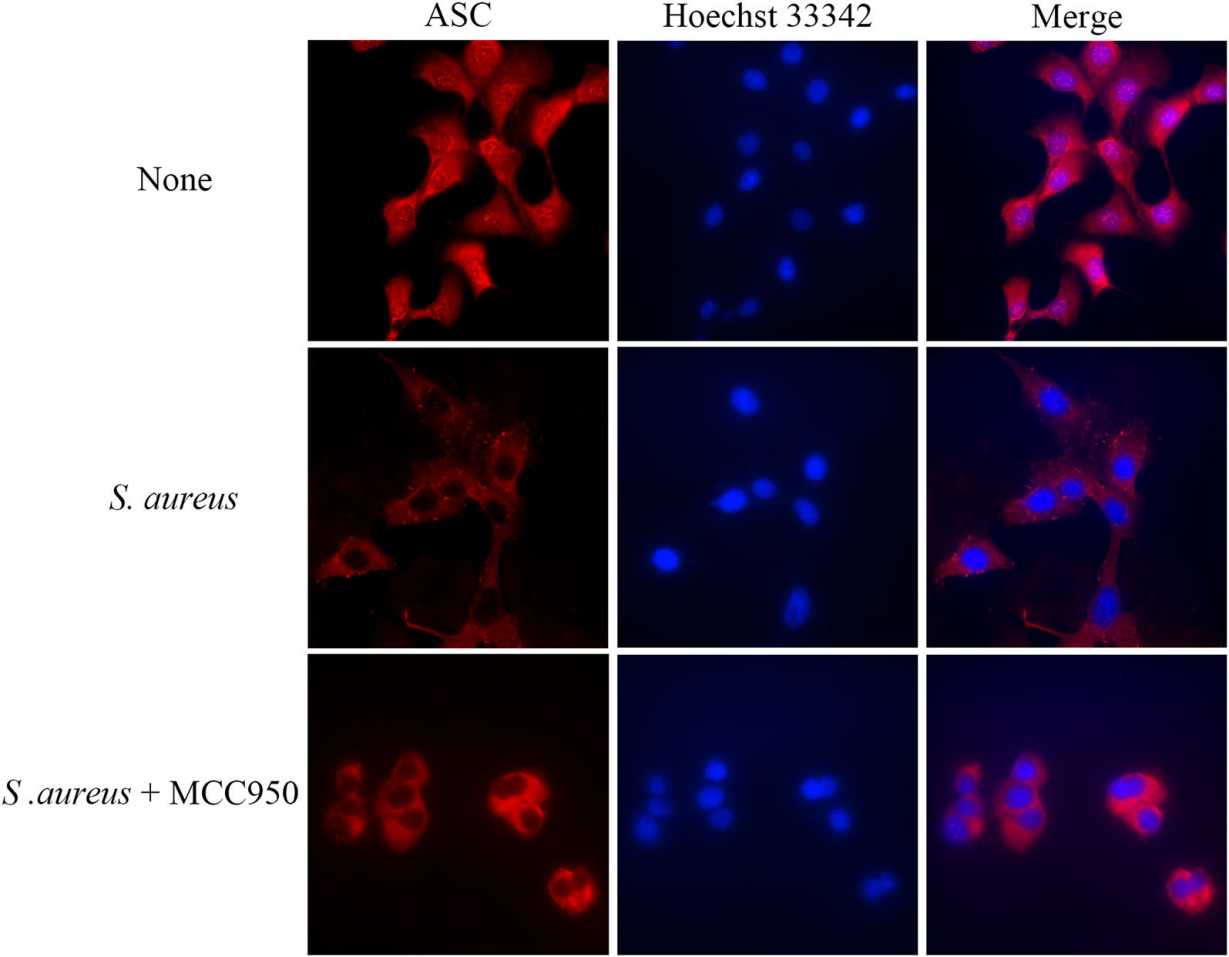
**NLRP3 inflammasome regulates ASC speck formation during *S. aureus*.** Fluorescence microscopy images of MAC-T cells immunoassayed for ASC (red) with or without MCC950 after 4 h of *S. aureus* treatment.

In addition to caspase-1 cleavage secretions of IL-18 and IL-1β, another marker of inflammasome activation is the formation of ASC specks. ASC specks were observed after *S. aureus* infection of MAC-T cells. ASC specks were significantly reduced when the cells were pretreated with the NLRP3 inhibitor MCC950 (Figure 4). Upon inflammasome activation, ASC is activated by NLRP3. Activated ASC is recruited from the nucleus to the cytoplasm and aggregates to form specks, which can be visualized by immunofluorescence microscopy [10, 11].

### NLRP3 inflammasomes and caspase-1 are essential for *S. aureus*-mediated release of IL-1β and IL-18 from MAC-T cells

**Figure 5:**
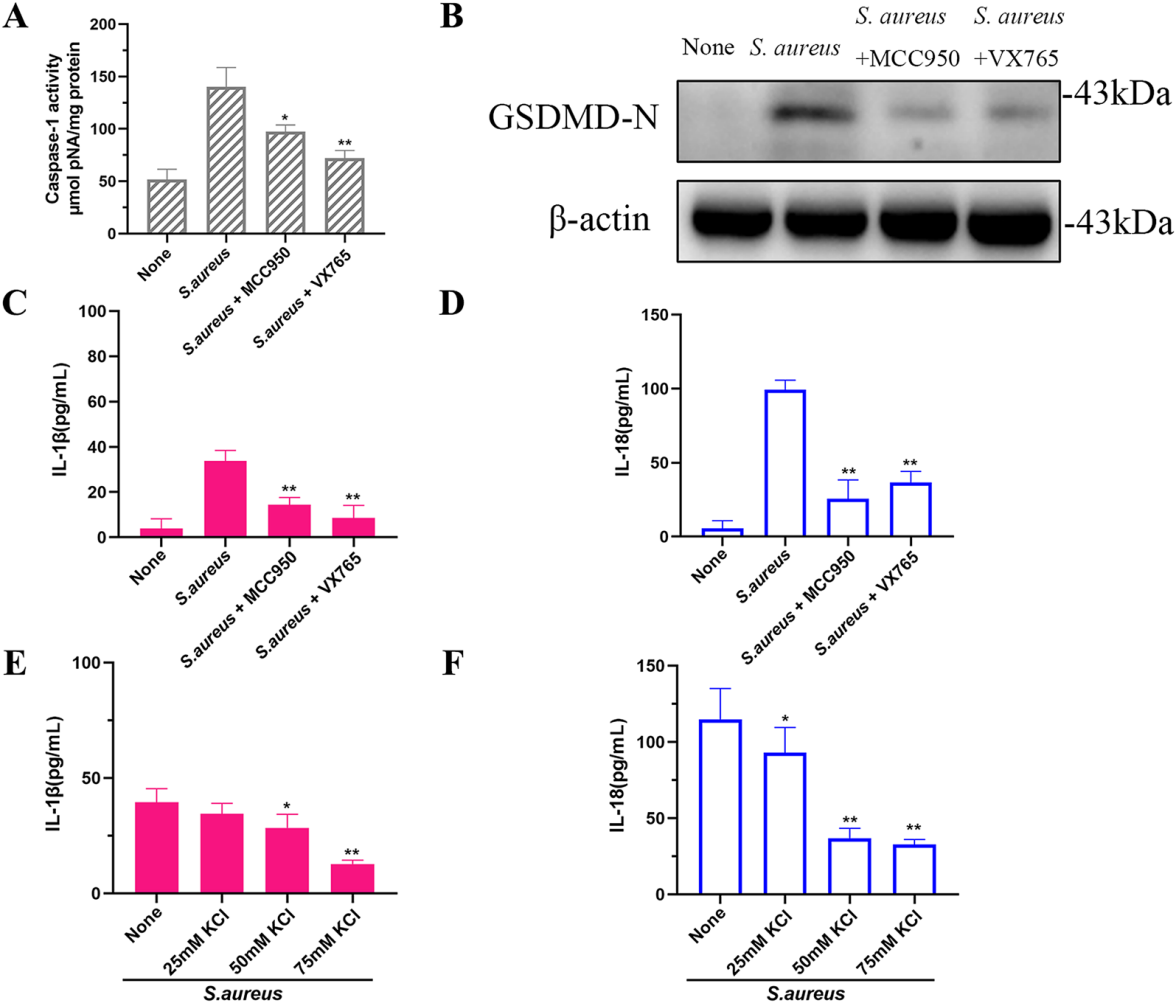
**NLRP3 inflammasome activation by** ***S. aureus*** **is essential for the generation of GSDMD-N and the release of IL-1β and IL-18.** **A** Activated caspase-1 and **B** GSDMD-N or released **C** IL-1β and **D** IL-18 of MAC-T cells treated with or without NLRP3 inhibitor MCC950 or caspase-1 inhibitor VX765 for 1.5 h prior to treatment with *S. aureus* for 4 h. **E** Levels of IL-1β and **F** IL-18 released after *S. aureus* treatment of MAC-T cells for 4 h in the presence or absence of 25, 50, or 75 mM KCl.

To assess the role of NLRP3 and caspase-1 in the cleavage of GSDMD caused by *S. aureus*, we pretreated cells with the NLRP3 inhibitor MCC950 or the caspase-1 inhibitor VX765. Inhibition of NLRP3 or caspase-1 in MAC-T cells reduced the activity of caspase-1 induced by *S. aureus*. GSDMD-N expression was also suppressed (Figures 5A and B). IL-1β and IL-18 release caused by *S. aureus* was significantly reduced when the cells were pretreated with MCC950 or VX765 (Figures 5C and D). To assess whether the induction of NLRP3 inflammasome activation by *S. aureus* was dependent on K^+^ efflux, MAC-T cells were pretreated with high concentrations of potassium chloride (KCl) for 1 h prior to *S. aureus* stimulation for 4 h. In MAC-T cells pretreated with KCl, a dose-dependent inhibition of *S. aureus*-mediated IL-1β and IL-18 release was observed. Moreover, caspase-1 activity was observed (Figures 5E and F). *S. aureus*-induced secretion of the pro-inflammatory cytokines IL-1β and IL-18 by MAC-T cells depended on NLRP3 inflammasome activation.

## Discussion

Bovine mastitis triggered by *S. aureus* infection remains a major problem in the dairy industry worldwide due to its pathogenicity, infectivity, colonization of skin or mucosal epithelium, persistence in the dairy environment, and poor therapeutic efficacy [19, 20]. Adhesion and invasion of bovine mammary epithelial cells are important factors in the formation of chronic infections [3]. *Staphylococcus aureus* can be ingested by bovine mammary epithelial cells, and intracellular *S. aureus* can escape endosomes and induce inflammation and cell death [21, 22]. Investigating the pyroptosis of MAC-T cells triggered by *S. aureus* contributes to the elucidation of the molecular basis of *S. aureus* mastitis pathogenesis.

Although apoptosis is the major form of regulated cell death, it is by no means the only form. A previous study described types of regulated cell death, including pyroptosis, necroptosis, and ferroptosis [23]. Pyroptosis, as an innate immune response to intracellular pathogens, is executed by caspase dependent cleavage of GSDMD [24]. In addition to caspase-1, caspase-11 can also mediate pyroptosis. Caspase-11 is caspase-1 independent and is activated by direct sensing of intracytoplasmic LPS [25]. In GSDMD-deficient cells, inflammasome stimulation induces apoptosis with the concomitant activation of caspase-3, which is largely caspase-1 dependent [26]. Inflammasomes are intracellular supramolecular complexes composed of a sensor molecule, ASC, and the effector caspase-1. When inflammasome sensor molecules are activated, ASC self-associates into helical fibrous assemblies, leading to the formation of the ASC speck [27]. The ASC speck acts as a molecular platform for the activation of pro­caspase-1. The NLRP3 inflammasome is a cytosolic signaling complex that mediates the activation of inflammatory mediators and can be activated by many danger signals closely associated with the pathogenesis of many common diseases [28–30]. Intracellular pathogens can activate NLRP3 inflammasome, causing the activation of inflammatory caspase-1, which in turn cleaves the pro-inflammatory cytokines IL-1 β and IL-18, leading to their maturation. The activated caspases-1 can cleave GSDMD into GSDMD-N and form pores in the plasma membrane through the aggregation of GSDMD-N fragments [31]. During pyroptosis, the GSDMD-N fragment permeabilizes the plasma membrane, leading to the release of IL-1β, IL-18, and LDH [5]. LDH is a cytosolic enzyme that is released into the culture medium when membrane integrity is lost [32]. Some factors that contribute to NLRP3 activation are K^+^ efflux, ROS production, lysosomal destabilization, and rupture [33–35].

*Staphylococcus aureus* mastitis is an inflammatory reaction of the mammary gland that causes damage to mammary tissue and epithelial cells, which further results in reduced milk synthesis and secretion [36]. *Staphylococcus aureus* can invade within bovine mammary epithelial cells and multiply before causing cell death, which aid in the establishment of recurrent subclinical infections [37]. NLRP3 inflammasome is an important signaling pathway of the innate immune system and is essential for host defense against bacterial infection [38]. Pyroptosis induced by bacteria through the activation of NLRP3 inflammasome causes rupture of the cell membrane and the release of cytokines, which leads to the release of intracellular bacteria and the recruitment of inflammatory cells [39]. Thus, pyroptosis in bovine mammary epithelial cells is instrumental for replication niche deprivation and clearance of *S. aureus* later in infection. However, inappropriate or excessive activation of pyroptosis in epithelial cells can also lead to tissue damage [40]. Pyroptosis as a double-edged sword that plays a critical role in antibacterial defense and tissue damage. In this study, we developed a GSDMD-N antibody that can be used to detect bovine species and demonstrated that *S. aureus* is sensed by the NLRP3 inflammasome in MAC-T cells. Similar to previous studies, this process could be blocked by the inhibition of K^+^ efflux [41]. Our data suggested that upon NLRP3 activation by *S. aureus*, ASC is recruited, and specks are formed, subsequently leading to the cleavage of caspase-1. Activated caspase-1 cleaves pro-inflammatory cytokines IL-1β, IL-18, and GSDMD. The generation of GSDMD-N allows it to oligomerize and translocate to the plasma membrane, thereby inducing cell rupture and the release of IL-1β and IL-18.

*Staphylococcus aureus* can activate NLRP3 and cause MAC-T cell pyroptosis via the K^+^ efflux pathway. Our work provides insight into the potential role of NLRP3 in the molecular pathogenesis of *S. aureus* mastitis and contributes to the elucidation of the molecular basis of *S. aureus* mastitis pathogenesis.

## Supplementary Information

**Additional file 1. Peptide sequence used for antibody preparation.**

## Abbreviations

*S. aureus*: *Staphylococcus aureus*
MAC-T: a cell line produced from primary bovine mammary alveolar cells by stable transfection with SV-40 large T-antigen
NLRP3: NOD-like receptor protein 3
ASC: apoptosis-associated speck-like protein
GSDMD: gasdermin D
LB: Luria–Bertani
DMEM: Dulbecco’s modified Eagle medium
FBS: fetal bovine serum
Ac-YVAD-pNA: Acetyl-Tyr-Val-Ala-Asp p-nitroanilide
pNA: p-Nitroanilide
MOI: multiplicity of infection
PI: propidium iodide
PS: phospholipid phosphatidylserine
