# Supplementary material for: Staphylococcus aureus mediates pyroptosis in bovine mammary epithelial cell via activation of NLRP3 inflammasome
Source: Vet Res. 2022 Feb 5;53:10. doi: 10.1186/s13567-022-01027-y (PMC8817610; doi:10.1186/s13567-022-01027-y)
Supplement: Supplementary file 1 — Additional file 1. Peptide sequence used for antibody preparation. [file 13567_2022_1027_MOESM1_ESM.docx]

The peptide sequence used for antibody preparation: masafekvvr svvreldhkd ltpvdslwss tsfqpytlls rkplssrfwr prykcvnlsi rdilepdape palecgrtfq fhdam
